# The Development and Initial Validation of a Short, Self-Report Measure on Social Inclusion for People with Intellectual Disabilities—A Transnational Study

**DOI:** 10.3390/ijerph18052540

**Published:** 2021-03-04

**Authors:** Piritta Asunta, Pauli Rintala, Florian Pochstein, Nelli Lyyra, Roy McConkey

**Affiliations:** 1 LIKES Research Center for Physical Activity and Health, 40700 Jyväskylä, Finland; piritta.asunta@gmail.com; 2 Faculty of Sport and Health Sciences, University of Jyväskylä, 40014 Jyväskylän, Finland; pauli.rintala@jyu.fi (P.R.); nelli.lyyra@jyu.fi (N.L.); 3 Faculty of Special Needs Education, University of Education Ludwigsburg, 71634 Ludwigsburg, Germany; pochstein@ph-ludwigsburg.de; 4 Institute of Nursing and Health Sciences, Ulster University, Newtownabbey BT37 0QB, UK

**Keywords:** intellectual disability, social inclusion, sports, community, self-report measures, transnational

## Abstract

Sport has been promoted as a means of increasing the social inclusion of persons with intellectual disabilities. Suitable tools for evaluating this claim are not readily available. The aim of this study was to develop a self-report tool for use by people with intellectual disabilities regarding the social inclusion they experience in sport and in the community. A three-phase process was used. In the first phase an item bank of questionnaire items was created and field-tested with 111 participants. Initial factor analysis identified 42 items which were further evaluated in Phase 2 with 941 participants from six European countries. Construct validity was established first through Exploratory and then Confirmatory factor analysis. These analyses identified ten items relating to inclusion in sports and ten to inclusion in local communities. A third phase checked the usability and test-retest reliability of the short form with a further 228 participants. In all, 1280 athletes and non-disabled partners were involved from eight countries. This short social inclusion questionnaire has been shown to be a reliable and valid measure for use transnationally. Further psychometric properties remain to be tested; notably its sensitivity to change resulting from interventions aimed at promoting social inclusion.

## 1. Introduction

People with intellectual disability often face social isolation [1]. The stigma and prejudices associated with this disability internationally has alienated them within their communities [2], even within their family circle [3]. In part, their exclusion is compounded by the specialist services provided to them and their families. Many continue to be segregated from their peers from early childhood through to old age as they attend special schools and centers away from those provided for the wider community [4]. Yet the United Nations (UN) Convention on the Rights of Persons with Disabilities [5] outlines a vision of their inclusion within society with its principles of “full and effective participation and inclusion within society.”

Article 30 of the Convention focuses on participation in sport and asserts that: “States Parties shall take appropriate measures … to encourage and promote the participation, to the fullest extent possible, of persons with disabilities in mainstream sporting activities at all levels”. To date, the inclusion in mainstream sports of people with disability has received little attention. Rather, the focus has been on special sports provision such as Paralympics [6]. 

People with intellectual disability face particular challenges in being included in sport [7]. Nonetheless Special Olympics (SO)—an international organization founded in 1968 and now present in over 170 countries globally—has shown the willingness and competence of persons with intellectual disability to train for and compete in, a variety of sports. Moreover, a growing body of research has evidenced its positive impact on athletes with intellectual disability [8]. But SO also views sport as a means to an end: namely to promote greater inclusion of people with intellectual disabilities through the medium of sport [9]. Latterly, SO has expanded their Unified Sports program that brings together players with and without intellectual disabilities on the same team, such as soccer, basketball and volleyball. The intention is to form relationships among the players that will spill over from the playing field into the wider community [10]. 

The present study was commissioned by SO to evaluate the impact of Unified Sports on athlete’s social inclusion. Current measures of social inclusion are not well suited for use with athletes with intellectual disabilities [11]. They do not take into account the communication difficulties experienced by people with intellectual disability; especially in terms of accessible language and the format of the ratings scales used [12]. Such adaptations are examples of the ‘reasonable adjustments’ required by disability discrimination legislation in many countries [5]. In any case, existing measures are not easily adapted to examine inclusion in a sports context [13]. 

The goal was to develop a questionnaire tool that could be used by SO in their programs worldwide to describe the extent of inclusion athletes experienced both within Special Olympics’ training activities and competitions and their inclusion in the wider community. Information gathered from the questionnaires could be used to assess and enhance the effectiveness of SOs in promoting social inclusion at a local level. Such a tool could also have wider use within mainstream sports as national governments seek to advance the aspirations of the UN Convention towards inclusive sports [14]. 

The measurement of social inclusion is especially challenging in a number of ways. To date, most of the literature has relied on proxy informants—family members and professionals—in assessing the social inclusion of persons with intellectual disability who are known to them. However, their reports may not accurately reflect the person’s own perceptions and experiences [15]. Hence our goal was to produce a self-report tool that captured the extent of inclusion experienced in sport by players who have an intellectual disability [16]. Their low levels of literacy and weaker communication skills would necessitate developing a questionnaire that was accessible to as wide a range of informants as possible [17]. 

A second major challenge was defining the concept of social inclusion. Although up to 10 possible measures have been developed on this theme, even the two most promising ones (the Activity and Participation Questionnaire (APQ-6) and the Social and Community Opportunities Profile (SCOPE)—short form) [11] were seen as not fit for the purposes as noted in the previous paragraph. In addition, the available social inclusion measures have undergone fairly limited psychometric assessment and none of the measures had been tested within a sports context [11]. The conceptual frameworks emerging around social inclusion for persons with intellectual disability tend to focus on two aspects—being connected into social networks and participation in community activities [18]. However, these broad domains need to be tailored to the specific context of sport. Our chosen solution was to first use a qualitative approach informed by phenomenology which had been identified as a feasible approach for persons with mild and moderate intellectual disabilities [19]. Our aim was to ascertain what social inclusion in sports means to players with an intellectual disability and likewise inclusion in local communities and neighborhoods. From the reports of their lived experiences, potential questionnaire items could be selected and constructed thereby ensuring the face and content validity of the intended measure in relation to what players with intellectual disability valued. Their insights would be especially useful for a tool intended to monitor meaningful comparisons and changes in social inclusion resulting from participation in sports. 

A third challenge was to ascertain if the tool could be used internationally and with athletes involved in traditional, segregated sports provided by SO as well as participants in Unified Sports. Many existing measures of social inclusion have been developed and tested solely within the one country and culture [11]. 

The aims of the present study were:

- To create an accessible self-report questionnaire for use with respondents who had an intellectual disability that described the social inclusion of players within sport and also within their local neighborhood or community.
- To assess the psychometric properties of the questionnaires relating to sports and to community inclusion with respondents drawn from different countries and translated from English into four other European languages.

## 2. Materials and Methods

### 2.1. Designing the Questionnaire

As noted above, the questionnaire was developed from a qualitative study that took place first with SO Unified Sports teams in the USA, Germany and India. Focus groups were held with over 80 athletes with intellectual disability and non-disabled sports partners to gain an insight into what social inclusion meant to both groups. This was asked in the context of SO Unified Sports and also about their inclusion in their local community or neighborhood. The main findings are summarized in a separate paper [20]. Additional focus groups were also held with 42 athletes with intellectual disability from four European Union (EU) countries—Finland, Poland, Germany and Austria—participating at the SO World Winter Games in Austria in 2017 [21]. 

The qualitative data from the focus groups identified a core theme of ‘togetherness’ as the underlying meaning of inclusion for the players in SO. Five subthemes were also apparent in the transcripts—equality (“all players at treated as equals”), communication, (“do other players listen to you”), friendship (“do you get invited to parties”), connections (“do you feel welcome when visiting local shops or cafes”), and assistance (“do other players comfort you and care for you). These subthemes are described in more detail in the referenced articles. For each subtheme, a series of statements relating to each was listed and these formed the initial corpus of items for use in the questionnaire. 

In the current study an advisory group of research partners who had been involved in leading the focus groups reviewed the items and selected those that were most frequently mentioned and most relevant to athletes involved with Special Olympics. This would ensure the face and content validity of the questionnaire items as well as confirming that meaning when translated in other languages. The items were grouped into two sections: those relating to sports and items describing inclusion in the local neighborhood or community. The items were mainly based on verbatim comments of players. 

**Figure 1:**
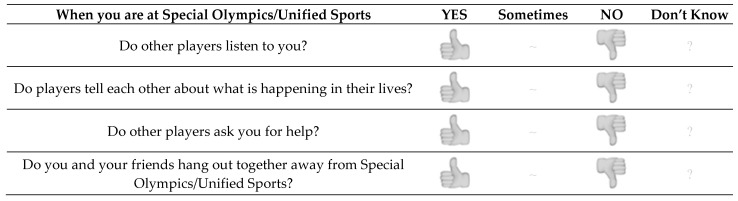
An example of questionnaire items.

A three-point rating scale was chosen for its simplicity: ‘Yes, Sometimes, No’ along with a ‘Don’t know’ option. Also visual referents were used to further clarify the available options and to allow for non-verbal responding [12]. An example of the questionnaire items is given in Figure 1.

A three-phase process was followed in refining the questionnaire items. The main features of each phase are summarized in Table 1. Each phase involved a new sample of respondents. 

Throughout all phases of the study, demographic information about the players was also gathered and refined. All were classed as intellectually disabled by Special Olympics but details of IQ and level of disability were not requested. They participated in various summer and winter sports, most commonly basketball, swimming, soccer, athletes, bowling, skiing and floorball.

### 2.2. Procedure

A favorable ethical opinion about the study was given by the Institutional Review Board of SO. An information sheet and consent form was given to all participants. This was translated into their local language in an easy-read format with illustrations. Throughout it was made clear that participation was voluntary and would not affect their involvement in SO, all answers were confidential and no one would be identified in any reports.

A network of researcher partners who had previously worked on other research and evaluation projects for Special Olympics was recruited from University personnel in Europe and the USA. A two-day planning workshop was held at the outset to plan the data gathering with a further workshop held towards the end of study to finalize the data analysis and conclusions. The research partners were fluent English speakers and undertook the translations of materials for their country as required. The translations were cross-checked and re-translated to English by the researchers who shared a common language.

Data gathering with athletes and non-disabled partners in Unified Teams took place mostly at training or competition events. University researchers (faculty staff and postgraduate students) individually interviewed athletes by reading the questionnaire items to the athletes and recording their answers either given verbally or by having them point to their choice on the rating scale that was provided. In addition, a small number of co-researchers with intellectual disability were recruited and trained to assist with the interviews in Germany and Poland. Questions were rephrased if respondents requested it or appeared by the interviewer not to understand [22]. The partners in Unified Sports team self-completed a paper version of the questionnaire. 

Further details of the participants, the statistical analyses undertaken and the findings are reported separately for each phase of the study in the following sections. 

## 3. Results

The development and field-testing of the questionnaire will be described in the three phases summarized in Table 1.

### 3.1. Phase 1: Preliminary Study

The initial questionnaire was first piloted with a Unified Sports Team in the USA and then more extensively during the SO Winter World Games in Austria in 2017. The purpose of the Phase 1 was to explore the construct validity and internal consistency of the questionnaire.

#### 3.1.1. Participants

The sample (*n* = 111) comprised athletes with intellectual disabilities from Finland (*n* = 34), Poland (*n* = 20), Germany (*n* = 30) and Austria (*n* = 27). In all, 78 (70%) were male. The mean age was 28.3 (SD 10.67) years and half (*n* = 55) were aged 20 to 29 years, with 19 (17%) under 20 years and 37 (33%) aged 30 years or over. Over two-thirds of the athletes (*n* = 75) had been involved with SO for four or more years with 25 persons (23%) involved for two or three years and 11 (10%) involved for one year or less.

#### 3.1.2. Measures 

The Initial item pool consisted of 29 items relating to Special Olympics sports and 28 items to community inclusion. These were drawn from the transcripts of the focus groups undertaken previously. 

#### 3.1.3. Statistical Analysis

Using the Statistical Package for Social Science (SPSS version 24), descriptive statistics were used to examine the proportion of respondents answering ‘Yes, Sometimes, and No’ as well as ‘Don’t know’ responses. Construct validity was established first through explorative factor analysis (EFA) and principal component analysis (PCA) with varimax rotation. Internal consistency was calculated using Cronbach’s coefficient alpha as a measure of internal reliability. It examines how well all the items measure the same construct. 

#### 3.1.4. Results 

First, a Principal Component Analysis was undertaken of all the items across the two sections. This confirmed that items relating to Sport loaded on different factors to those relating to inclusion in local neighborhoods. In addition, separate Principal Component analyses identified items in each section that loaded highest on each of the factors with an eigenvalue greater than 1 with reasonable internal reliabilities on each factor. Further details of these analyses are available on request.

A common comment from the interviewers was that the questionnaires needed to be shorter to maintain the interest and attention of the respondents. Thus 21 items in each section were identified for field-testing and selected on the basis of factor loadings and the spread of responses obtained in Phase 1. 

### 3.2. Phase 2: Field-Testing

The revised questionnaire was then field-tested with Unified teams in the USA and with participants in traditional SO and Unified Sports participants in six EU countries; both athletes and non-disabled partners (see Table 1). The purpose was to determine the construct validity and internal consistency with larger sample and the revised questionnaire.

#### 3.2.1. Participants

*Athletes with intellectual disabilities*: Of the 788 athletes, 579 participated in traditional (segregated) Special Olympics and 209 in Unified Sports. In all, 474 (60%) were male and 270 (34%) were aged under 20 years; with 277 (35%) aged 20 to 29 years and 235 (30%) aged 30 years or over. Their mean age was 25.6 years (SD 10.41). Sixty-one percent of the athletes (*n* = 484) had been involved with Special Olympics for four or more years with 123 persons (16%) involved for two or three years and 168 (22%) involved for one year or less.

*Non-disabled partners in Unified Sports*: Of the 153 partners, 85 (55%) were male and 62 (41%) were aged under 20 years; with 58 (38%) aged 20 to 29 years and 31 (21%) aged 30 years or over with a mean age of 23.2 years (SD 11.12). Twenty-nine percent (*n* = 45) had been involved with Unified Sports for four or more years with 53 persons (35%) involved for two or three years and 54 (36%) involved for one year or less.

#### 3.2.2. Statistical Analysis

Principal Component Analyses (PCA) were undertaken to identify the items loadings and communalities. Kaiser-Meyer- Olkin (KMO) measures and Bartlett’s sphericity test showed that the data met the criteria for factor analysis. Confirmatory Factor Analyses (CFA) of the questionnaire items were then undertaken using MPLUS version 7.0 and were based on the preliminary PCA and themes identified in the earlier qualitative study as noted in the introduction. For the confirmatory factor analyses, the parameters were estimated by maximum likelihood robust (MLR) estimation method. Missing values were treated with missing at random (MAR) procedure in Mplus. However, the answer option ”I Don’t know” was excluded from the analyses although this response was rarely chosen. 

Factor analysis of the two sets of items combined had again confirmed two factors: one relating to sports and the second to community inclusion. Consequently, separate confirmatory factor analyses were undertaken for the 21 items relating to sports and for the 21 items relating to community. The model fit in CFA was evaluated using the comparative fit index (CFI), the Tucker-Lewis index (TLI), the root mean square error approximation (RMSEA) and the standardized root mean square residual (SRMR). The following cut-off values indicating a good fit were applied: CFI, TLI > 0.95; RMSEA < 0.06; SRMR < 0.08 [23]. These analyses, allied to the descriptive statistics of item responses, were used to identify a shorter 10 item questionnaire suitable for use with persons with intellectual disabilities.

#### 3.2.3. Results

*Inclusion in sport:* The results showed that the KMO value for the sport inclusion items was 0.731, and the significance of Bartlett’s sphericity was *p* < 0.001 (χ^2^ = 734.47, df = 45, *p* < 0.001). The four factors estimated by PCA accounted for 58.4% of the variance. These factors were confirmed in the CFA with good fit statistics with athletes with ID (*n* = 788; CFI = 0.94, TLI = 0.90, RMSEA = 0.03, SRMR = 0.04) and when athletes and partners were combined (*n* = 941; CFI = 0.95, TLI = 0.92, RMSEA = 0.03, SRMR = 0.03).

The four factors relating to athlete’s perceptions of their inclusion in sports and the factor loadings of items measuring these four factors are summarized in Table 2 and the items are described later in Table 4. 

*Community Inclusion:* For the items relating to community inclusion, similar analyses were undertaken. For the community items the KMO value was 0.72, and the significance of Bartlett’s sphericity was *p* < 0.001 (χ^2^ = 1354.03, df = 45, *p* < 0.001). The four factors estimated by PCA accounted for 64.8% of the variance and the CFA confirmed good structural validity with athletes (CFI = 0.99, TLI = 0.99, RMSEA = 0.02, SRMR = 0.02) and also when the analyses were repeated with partners added (CFI = 0.99, TLI = 0.98, RMSEA = 0.02, SRMR = 0.02). The four factors and the loadings of items measuring the four factors are summarized in Table 3 and the items are named in the following Table 5. 

In sum, the four factor models with 10 items in both sections indicated good fit and the model is consistent with the data. The structure was equally good when the partners were included in the analyses. Other models were tested, but the above structures were the best-fitting model and had the best internal consistency values. The Cronbach’s alpha value across all 20 items together was 0.75. For the 10 items relating to Sports Inclusion, the Cronbach’s alpha was 0.64 (both with and without partners) and for the 10 community inclusion items, the Cronbach’s alpha was 0.64 with only athletes and 0.63 with athletes and partners. However, these lower values are not unexpected given the four factors identified in each analysis [24]. 

### 3.3. Phase 3: Usability, Reliability and Predictive Validity

In Phase 3, the revised questionnaire of 10 items relating to sports inclusion and 10 items of community inclusion, was further checked for ease of administration with players from a wider range of countries along with test-retest reliabilities and with naïve interviewers. Table 1 summarizes the participants in this Phase who were involved in Unified Sports and were recruited mainly at an International event for Unified teams organized by Special Olympics in 2018. Interpreters were provided as needed. 

At this stage, two practice items were included at the start of the questionnaire to further ensure that athletes in particular understood the rating scale [25]. The items were: “Do you look forward to going to Special Olympics/Unified Sports? And “Do you feel left out at Special Olympics/Unified Sports?” In the field-testing these items had nearly 100% responses ‘Yes’ to the first question and 100% ‘No’ to the second. A copy of the final scale is given as a Supplementary File. 

The test-retest reliability of the short questionnaire for athletes and partners was assessed with a subset of 63 players (34 athletes and 29 partners) of Unified Teams (mainly basketball, soccer and handball) who completed the questionnaire on two separate occasions. This data was collected during the German National Games, and the time between the two points was 3 (minimum) to 5 (maximum) days.

Copies of the translated questionnaire in German, Finnish, Polish and Romanian are available on request from the corresponding author. 

#### 3.3.1. Participants 

*Athletes with intellectual disabilities:* Of the 118 athletes, 85 (72%) were male and around over half (*n* = 63) were aged under 20 years; with 47 (40%) aged 20 to 29 years and 7 (6%) aged 30 years or over with a mean age of 20.5 years (SD 5.52). Just under one-third of the athletes (*n* = 36) had been involved with Special Olympics Unified Sports for four or more years with 32 persons (27%) involved for two or three years and 50 (42%) involved for one year or less.

*Non-disabled Partners in Unified Sports*: Of the 110 partners, 65 (59%) were male and 63 (58%) were aged under 20 years; with 42 (39%) aged 20 to 29 years and 4 (4%) aged 30 years or over, with a mean age of 19.7 years (SD 4.38). Over half of the partners (*n* = 64) had been involved with Unified Sports for four or more years with 29 persons (26%) involved for two or three years and 15 (14%) involved for one year or less.

#### 3.3.2. Results 

The shortened questionnaire was successfully completed by both athletes with intellectual disabilities and non-disabled partners in around 10 min with few difficulties reported. There were few problems with interpretation into other languages. 

Test-retest reliability was checked in two ways. When responses on the two occasions were compared, the average percentage agreement in responses across the ten items for sports inclusion was 89% for athletes and 93% for partners. For the community inclusion items there was 86% agreement for athletes and 94% agreement for partners. 

A summary score was also calculated; namely, a count of the 10 items that had been answered ‘Yes’ at both time points. The Pearson Product Moment Correlation for athletes’ scores on the sports inclusion items was r = 0.87 and for partners it was r = 0.89. The equivalent correlations for the community inclusion items for athletes was r = 0.85 and for partners it was r = 0.95. There were no statistically significant difference between the mean scores on test and retest for both athletes and partners. Hence, with this sample of 63 Unified Sports athletes and partners, the questionnaire had good test-retest reliability, with partners tending to provide more consistent responses than athletes.

#### 3.3.3. Predictive Validity 

As well as establishing the face and content validity of the questionnaire, its predictive validity was explored by comparing the responses of three groups—athletes with intellectual disabilities involved with traditional Special Olympics; athletes involved in Unified Sports and non-disabled partners involved in Unified Sports. Based on past research, it would be expected that partners would report greater social inclusion than athletes at least in terms of community inclusion. Also, athletes may report greater inclusion in sports than in the community [1]. 

Table 4 and Table 5 summarize the percentage of players answering ‘Yes’ to each item within the final version of the questionnaire. These percentages are based on all participants who responded to these items during all stages of the questionnaire development. Chi Square tests were used to test the significance of differences across the ratings: ‘Yes: Sometimes: No’. In all but two of the 10 sports items there were statistically significant differences across the three groupings but with the community inclusion items all differences were significant (*p* < 0.001). 

In addition, a summary score could be calculated for each section of the questionnaire by counting the number of items answered ‘Yes’ by respondents giving a range of scores from 0 to 10 (high inclusion). For this analysis two items in the community section were reversed coded—items 2.9 and 2.10—in which a NO response was indicative of higher inclusion. Table 6 presents the mean scores (SDs) for inclusion in sports and in the community for the three groups. 

The Pearson Product moment correlation between scores on sports and community inclusion across the two athlete groups (*n* = 1.017) was r = 0.47 which although statistically significant, the amount of shared variance is small at 22%. For Unified Sport partners, the correlation was r = 0.32 with even less shared variance.

When the scores across the three groups were compared, partners had significantly higher scores in terms of community inclusion but less so for sports inclusion. The differences between the two groups of athletes on both sport and community inclusion were not significant (post-hoc Tukey tests; *p* > 0.01). 

## 4. Discussion

This study has a number of unique strengths. The questionnaire is based around the lived experiences of athletes with intellectual disability from different countries around the world. It explores both their inclusion within a variety of sports as well as in their local community or neighborhood. An iterative process was used in developing the questionnaire which was tailored to their particular needs as respondents with intellectual disabilities, especially with respect to the question wording, rating scale and number of questions asked. The items were mostly in plain English and were readily translated into other European languages: German, Finnish, Polish and Romanian. The questionnaire has reasonable psychometric properties in terms of reliability and validity. 

The study confirms that people with intellectual disability can be reliable informants. Previous studies have shown that their perceptions are often different to those of proxy informants such as family carers and teachers [15]. Thus, the social inclusion questionnaire provides a useful tool for use in future research within and beyond sports in which people with intellectual disabilities are informants, notwithstanding that the views of coaches—among others—could also be sought. Indeed, a parallel project to this one with athletes, has resulted in a questionnaire for use with coaches [26]. 

A number of features of the questionnaire are worth highlighting. The three points rating scale, which was chosen for its simplicity, worked effectively when gathering the responses and reasonably well in data analysis. On reflection though, the terms ‘Yes’ or ‘No’ seemed too definite and might be better replaced by an indication of frequency: for example: ‘Nearly always: Sometimes: Rarely or Never’. However, the symbols of thumbs up or down could be retained as these were arguably more meaningful to the respondents especially those with more marked communication difficulties. 

A possible disadvantage of such simple ratings is the lack of sensitivity when using the questionnaires to detect change over time or when making comparisons across different sub-groupings of athletes, for example those in Unified Sports and those in segregated sports. Nonetheless the summary scores developed from the two sets of ten items, did cover the full range of scores, even though in these samples they were skewed towards higher scores that were reflective of greater inclusion. That said, the differences among the subgroupings were small, albeit they were statistically significant. A more sensitive measure might emerge from a longer form of the questionnaire: a point we will return to later.

An interview format was deemed necessary for athletes with an intellectual disability given the lower level of literacy skills within this population [25]. The use of independent interviewers helped to ensure consistency of presentation and should have reduced the possibility of socially desirable responding which might have been an issue if coaches conducted the interviews, for example. Looking to the future, it is possible that electronic delivery of the questionnaires could be a route to self-completion by athletes. The software exists for the questions to be read to the respondent through pre-recorded audio with simple visual cues provided for their responses on either tablets or smart phones. This format might be preferable when the questionnaire is used by practitioners such as sports coaches, given that online delivery is thought to reduce socially desirable responding [27].

The relatively low internal reliability of items within the sports inclusion and the community inclusion sections is not unexpected given the different domains identified initially in the qualitative responses and confirmed in the factor analyses of the questionnaire data. Indeed, the literature affirms that the concept of social inclusion is not easily reduced to a single indictor [18]. However, reconciling the complexity of the social inclusion with a short, easily administered scale requires compromises. The use of summary scores for each factor identified in the factor analyses was not feasible given the small number of items within each one factor. Instead, we chose to select items that were most representative of the four factors. A total score across the items could still be interpreted as an overall indicator of the extent of a person’s inclusion. In this respect reliance on a high alpha score of internal consistency is not appropriate and indeed low alpha scores may underestimate the reliabilities of questionnaires [24]. Moreover, Nunnally [28] notes that new measures can be accepted with Cronbach’s alpha of 0.60. 

A further option would be to create a longer form of the scale by increasing the number of items within each factor and especially for those factors for which there are only two items. Additional items could come from those included in phases 1 and 2 of this study although the choice of further items may need to go beyond the items originally identified through the qualitative phase and draw on the wider intellectual disability literature and further indicators of social inclusion relating to each of the particular factors [15]. That said, it is debatable whether or not a profiling approach would be relevant to practitioners who are more likely to use generic strategies to promote inclusion across groups of players whereas a profile approach is often better suited to identifying and addressing individual variations in a person’s inclusion experiences [18]. Future use of the questionnaires would help to elucidate these issues. 

The availability of the questionnaire enables further tests of its validity to be undertaken. Of particular interest is its use to assess changes in social inclusion, notably those resulting from interventions aimed at promoting the inclusion of players with intellectual disability in mainstream sports and the spill-over into greater community inclusion away from the sports field. The latter is facilitated by having two sets of items; either one of which could be used separately if necessary. 

Our findings suggest that inclusion in the domains of sport and community is only weakly related both for players with intellectual disability and for non-disabled partners. This suggests that the measurement of inclusion in one area will not necessarily reflect a change in the other. Moreover, we have started to investigate how inclusion of students with intellectual disabilities in schools compares with their inclusion in sports by using the same item set in the context of schools as for sports. Moreover, both these contexts can be compared with respondents’ community inclusion [29]. 

The questionnaire was tested with men and women from a range of ages. Additional demographic information also needs to be gathered to enable possible predictors of inclusion to be identified. Past literature suggests this should include the person’s living arrangements, their level of functioning, the availability and use of public transport. Further studies have been undertaken recently to examine the predictors of inclusion in both sports and community settings [30]. 

Finally, we acknowledge some potential limitations to the study. Our samples were drawn from athletes who had greater opportunities to be included, especially within sports and Special Olympics in particular. In part this was deliberate as our intention was to discover what inclusion meant to these players. However, the use of the questionnaires with people who have limited opportunity to be included in sport or community settings remains to be tested. Also, the questionnaires contain few items related to social exclusion which may be the reality in lives of the wider population of people with intellectual disability [1]. 

Future studies might also examine the extent of inclusion across different sporting contexts, such as team sports versus individual sports. It should be replicated also in settings other than SO. Additionally, within the sports context, the items do not distinguish between inclusion among groupings of people with intellectual disability and the inclusion of these athletes with non-disabled peers. This distinction is of particular relevance when used in the context of Unified Sports when there is the risk that players with intellectual disability become a grouping apart from their non-disabled peers in the sports context. A similar argument applies also to community settings. However, the advisory panel for the study felt that terminology to distinguish the two sets of players would emphasize the difference among players which was contrary to the ethos of team building in Unified Sports. We also gained the impression that athletes in Unified Sports did not readily distinguish between players with and without disabilities. Nonetheless studies that wanted to focus on inclusion of people with intellectual disability with non-disabled persons solely, may need to adapt the wording of the questions to make this clear. 

## 5. Conclusions

Social inclusion questionnaires can be considered a quick and promising measure to give information on the extent of inclusion athletes with intellectual disabilities experience in sports and local communities. The questionnaires have reasonable internal reliability and good test-retest reliability. Evidence for their content, construct and predictive validity is presented. They could be applicable beyond sports and inevitably, further refinements will be identified through the use of the questionnaires by practitioners and researchers. Further psychometric properties of the measure remain to be tested; notably its sensitivity to change resulting from interventions aimed at promoting social inclusion.

## Figures and Tables

**Table 1:** Descriptive information of the three phases of questionnaire development.

Phases	I	II	III
Number of questionnaire items (SO ^1^ + Community ^2^)	29 + 28	21 + 21	10 + 10
Total number of participants	111	941	161
Athletes Special Olympics ^3^		579	0
Athletes Unified Sports ^4^	111	209	96
Partners Unified Sports ^5^		153	65
Countries involved	Austria, Finland, Germany, Poland	Finland, Germany, Ireland, Malta, Poland, Romania, USA	USA, Germany, Canada, Slovakia, Nigeria, Kenya, Mexico, Brazil, Uruguay, Korea
Statistical analyzes	EFA ^6^, Cronbach’s alpha	PCA ^7^, CFA ^8^, Cronbach’s alpha	Pearson Correlation and *t*-tests
Psychometric properties	Construct validity, internal consistency	Construct validity, internal consistency	Test-retest reliability, predictive validity
Timeline	spring 2017	autumn 2017	spring 2018

Notes: ^1^ Items relating to Special Olympics sports; ^2^ Items relating to community inclusion. ^3^ Participants in segregated sports provided by SO; ^4^ Athletes with intellectual disabilities in Unified Sports ^5^ Non-disabled partners of athletes with intellectual disabilities. ^6^ Exploratory Factor Analysis. ^7^ Principal Components Analysis. ^8^ Confirmatory Factor Analysis.

**Table 2:** Standardized item loadings for Confirmatory Factor Analyses the four factors relating to sports inclusion and item communalities for athletes with ID and for both athletes and partners *.

Item Number	Connection	Equality	Friendship	Assistance	h2	h2 *
1.1	0.629/0.632 *				0.60	0.58
1.2	0.606/0.621 *				0.52	0.53
1.3	0.437/0.478 *				0.48	0.50
1.4		0.298/0.251 *			0.61	0.67
1.5		0.589/0.582 *			0.53	0.50
1.6		0.247/0.246 *			0.52	0.53
1.7			0.498/0.485 *		0.43	0.43
1.8			0.450/0.482 *		0.35	0.31
1.9				0.467/0.503 *	0.49	0.51
1.10				0.555/0.561 *	0.40	0.39

Note: Factor loadings (CFA). Communality (PCA). Values are based on athletes data (*n* = 788)/* Partners and athletes included in the analysis (*n* = 1052). The items are named in Table 4.

**Table 3:** Standardized item loadings for Confirmatory Factor Analyses (CFA) and the four factors relating to community inclusion and item communalities for athletes with ID and for both athletes and partners *.

Item Number	Friendship	Assistance	Acceptance	Exclusion	h2	h2 *
2.1	0.692/0.704 *				0.60	0.61
2.2	0.724/0.738 *				0.55	0.57
2.3	0.774/0.792 *				0.72	0.72
2.4		0.681/0.681 *			0.46	0.49
2.5		0.793/0.797 *			0.72	0.73
2.6		0.771/0.765 *			0.68	0.65
2.7			0.670/0.622 *		0.41	0.39
2.8			0.729/0.709 *		0.49	0.49
2.9				0.526/0.500 *	0.49	0.50
2.10				0.503/0.501 *	0.42	0.42

Note: Factor loadings (CFA). Communality (PCA). Values are based on athletes data (*n* = 788)/* Partners and athletes included in the analysis (*n* = 1052). The items are listed in Table 4.

**Table 4:** The percentage of athletes and partners answering ‘Yes’ to the Sports Inclusion Items.

Sports Inclusion Items	Athletes SO (*n* = 690)	Athletes Unified (*n* = 327)	Partners Unified (*n* = 263)	Total
Factor 1: Connection				
1.1 Do you and your friends from Special Olympics hang out together away from Special Olympics/Unified Sports?	53.1%	49.8%	48.1%	51.3%
1.2. Do you often get invited to parties or other celebrations from people in Special Olympics/Unified Sports?	49.4%	38.5%	41.2%	45.0%
1.3. Do the players at Special Olympics/Unified Sports keep in touch with you by phone or text or Facebook?	61.4%	61.0%	59.4%	60.9%
Factor 2: Equality				
1.4. Does everyone get a chance to play at Special Olympics/Unified Sports?	89.6%	85.2%	83.5%	87.2%
1.5. Do your team mates trust you to play well at Special Olympics/Unified Sports?	91.0%	92.0%	93.4%	91.8%
1.6. Are all players treated as equals at Special Olympics/Unified Sports?	84.0%	90.7%	88.2%	86.6%
Factor 3: Communication/Friendship				
1.7. Do other players listen to you at Special Olympics/Unified Sports?	73.1%	73.2%	85.6%	75.8%
1.8. Do players tell each other about what is happening in their lives?	52.9%	43.8%	61.2%	52.3%
Factor 4: Assistance				
1.9. Do other players ask you for help at Special Olympics/Unified Sports?	54.5%	60.6%	77.1%	60.7%
1.10. Do other players comfort you and care for you at Special Olympics/Unified Sports?	79.2%	84.4%	82.3%	81.4%

Note: Don’t know responses were included in the calculating the percentages: Missing data was mostly less than 1% for each item. Chi Square tests of responses across the three groups were significant at *p* < 0.01 with the exception of questions 1.5 and 1.6.

**Table 5:** The percentage of athletes and partners answering Yes to the Community Inclusion Items.

Community Inclusion Items	Athletes SO (*n* = 690)	Athletes Unified (*n* = 327)	Partners Unified (*n* = 263)	Total
Factor 1: Friendships				
2.1. Do friends from the local community come over to your house? (other than family members)	48.8%	50.2%	76.6%	54.8%
2.2 Do you often get invited to parties or celebrations from people in your community? (Other than family members)	53.8%	47.8%	75.2%	56.7%
2.3 Do your friends invite you to hang out at their houses or in the community? (Other than family members)	52.2%	55.6%	80.5%	58.9%
Factor 2: Assistance				
2.4 Do you often talk with your neighbors: people living near your home?	69.2%	54.7%	67.6%	65.2%
2.5 Do your neighbors help you if you require help?	72.4%	63.8%	71.5%	70.2%
2.6 Do you help your neighbors if they require help?	78.9%	71.9%	85.3%	78.4%
Factor 3: Acceptance				
2.7 Do you feel you are an important member of your local community: do people want you to be there?	73.9%	70.4%	70.8%	72.4%
2.8 Do you feel welcome when visiting local shops or cafes?	82.5%	78.4%	87.0%	82.4%
Factor 4: Exclusion				
2.9 Do you often feel lonely and left out?	16.6%	13.5%	5.0%	13.5%
2.10 Do other people in your community call you bad names or mock you?	9.4%	8.4%	6.1%	8.5%

Don’t know responses were included in the calculating the percentages. Missing data was mostly less than 1% for each item. Chi Square tests of responses across the three groups were significant at *p* < 0.001.

**Table 6:** The mean and standard deviations for a count of ‘Yes’ ratings for the athletes and partners.

	Athletes SO (*n* = 690)	Athletes Unified (*n* = 327)	Partners Unified (*n* = 263)	Statistical Tests
Sports Inclusion	6.74 (*SD* 2.21)	6.66 (*SD* 2.23)	7.14 (*SD* 2.06)	F(2, 1277) = 7.14; *p* < 0.05
Community Inclusion	6.65 (*SD* 2.33)	6.21 (*SD* 2.70)	7.75 (*SD* 2.10)	F(2, 1277) = 31.99; *p* < 0.001

## Data Availability

The data reported in this study is available on reasonable request to the corresponding author.

## Supplementary Materials

The following are available online at https://www.mdpi.com/1660-4601/18/5/2540/s1, Copy of the final version of the questionnaire.
