# Supplementary material for: The Development and Initial Validation of a Short, Self-Report Measure on Social Inclusion for People with Intellectual Disabilities—A Transnational Study
Source: Int J Environ Res Public Health. 2021 Mar 4;18(5):2540. doi: 10.3390/ijerph18052540 (PMC7967343; doi:10.3390/ijerph18052540)
Supplement: Supplementary file 1 [file ijerph-18-02540-s001.pdf]

Social Inclusion - Self-report Questionnaires

I want to ask you some questions about social inclusion. Social inclusion means being together with other people. We want to find out how included you feel when you are with other people while playing sports and when in the place where you live - your neighbourhood and local community.

I have a list of questions we want you to answer (show next page). There are no right or wrong answers.

- If your answer to a question is *Yes* –we will put an X in the YES column.
- If your answer to a question is *No* – we will put an X in the NO column.
- If your answer to a question is *Sometimes* – then we will put an X in the SOMETIMES ~ column.
- If you *don't know* or *don't understand a question*, we will put an X in the DON'T KNOW ? column.

I will ask you the questions and you tell me your answer. We will keep your answers private. Many thanks for your help.

Notes to interviewers

The first two items in Section 1 are included as practice items. You can double check the answer the person gives by asking: Are you sure that is your answer? Why did you choose that? Praise the person for giving the answer. But only do this for the first two questions.

For athletes from traditional Special Olympics, omit mention of Unified Sports.
For athletes in Unified Sports, start by saying 'Special Olympics Unified Sports' for the first three items in Section 1, thereafter you can say 'Unified Sports'.

In calculating a total score add, up the number of items rated as YES although there is a slight difference in section 3: Community Inclusion.

© Special Olympics International, 2020.

Section 1: Special Olympics/Unified Sports

1. Do you take part in Unified Sports? Yes ◯ No ◯
   Are you: An athlete ◯ A Partner ◯ Assistant Coach/Volunteer ◯ Other ◯
   What sports do you play? ____

2. Do you take part in traditional Special Olympics? Yes ◯ No◯
   What sports do you play? ____

I would like to ask you some questions about Special Olympics/Unified Sports.

	YES	Some-times	NO	Don't Know
Do you look forward to going to Special Olympics/ Unified Sports?	👍	~	👎	?
Do you feel left out at Special Olympics/ Unified Sports/Unified Sports?	👍	~	👎	?
Do other players listen to you at Special Olympics/ Unified Sports?	👍	~	👎	?
Do players tell each other about what is happening in their lives?	👍	~	👎	?
Does everyone get a chance to play at Special Olympics/Unified Sports?	👍	~	👎	?
Do your team mates trust you to play well at Special Olympics/Unified Sports?	👍	~	👎	?
Do other players ask you for help at Special Olympics/Unified Sports?	👍	~	👎	?
Are all players treated as equals at Special Olympics/Unified Sports?	👍	~	👎	?
Do you and your friends from Special Olympics hang out together away from Special Olympics/Unified Sports?	👍	~	👎	?
Do you get invited to parties or other celebrations from people in Special Olympics/Unified Sports?	👍	~	👎	?
Do the players at Special Olympics/Unified Sports keep in touch with you by phone or text or Facebook?	👍	~	👎	?
Do other players comfort you and care for you at Special Olympics/Unified Sports?	👍	~	👎	?

Section 2: Your school/center/college.

These questions are about being included in your school. If you do NOT attend school or college then skip this section and go to Section 3

Do you attend school, a centre or a college? Yes ◯ No

Is it?: A special school or center. Yes ◯ No ◯

	YES	Some-times	NO	Don't Know
Do other students listen to you at your school?		~		?
Do students tell each other about what is happening in their lives?		~		?
Does everyone get a chance to take part at your school?		~		?
Do other students trust you to do well at your school?		~		?
Do other students ask you for help at your school?		~		?
Are all students treated as equals at your school?		~		?
Do you and your friends from school hang out together away from school?		~		?
Do the students at your school keep in touch with you by phone or text or Facebook?		~		?
Do you get invited to parties or other celebrations from students in your school?		~		?
Do other students comfort you and care for you at your school		~		?

Section 3: Local Community

These questions are about when you are at home in your neighbourhood and local community. Here we mean people and friends that do NOT take part in Special Olympics/Unified Sports.

	YES	Some-times	NO	Don't Know
Do other people in your community call you bad names or mock you?*		~		?
Do friends from the local community come over to your house? (other than family members)		~		?
Do you get invited to parties or celebrations from people in your community? (Other than family members)		~		?
Do your friends invite you to hang out at their houses or in the community? (Other than family members)		~		?
Do you talk with your neighbours: people living near your home?		~		?
Do your neighbours help you if you require help?		~		?
Do you help your neighbours if they require help?		~		?
Do you feel you are an important member of your local community: do people want you to be there?		~		?
Do you feel welcome when visiting local shops or cafes?		~		?
Do you feel lonely and left out?*		~		?

*for these items, a NO response is scored as 1 to obtain a total score with other items scored 1 for YES.

Can you tell me more about you?

Q1 What country do you live in? ______________________

Q2 What is your age? _______________ years

Q3 What is your sex?

- ❍ Male
- ❍ Female
- ❍ Don't know

Q4 Where do you live?

- ❍ With my parents
- ❍ With my partner such as wife/husband/
- ❍ On my own
- ❍ With roommates in a shared apartment or house
- ❍ In a group home or residential facility
- ❍ Other

Qu4b Do you live in?

- ❍ A large city
- ❍ A town
- ❍ In the country
- ❍ Not sure

Q5 Do you have a paid job?

- ❍ Yes
- ❍ No
- ❍ Don't know

Q6 Do you currently attend school, college or university?

- ❍ Yes
- ❍ No
- ❍ Don't know

Q7 How would you describe your health?

- ❍ Excellent
- ❍ Very good
- ❍ Fairly good
- ❍ Poor
- ❍ Don't know

Q8 How long have you been in Special Olympics/Unified Sports?:

- ❍ Joined last year/this year (2017/18)
- ❍ For two or three years
- ❍ For many years (four or more years)

Q9 Do you go on public transport such as bus, train or tram by yourself?

- ❍ Yes
- ❍ No
- ❍ None available
- ❍ Not sure

Q10 Do you use a mobile phone to talk to your family and friends?

- ❍ Yes
- ❍ No
- ❍ None available
- ❍ Not sure

Many thanks for answering my questions.
